# Genomic expression during human myelopoiesis

**DOI:** 10.1186/1471-2164-8-264

**Published:** 2007-08-03

**Authors:** Francesco Ferrari, Stefania Bortoluzzi, Alessandro Coppe, Dario Basso, Silvio Bicciato, Roberta Zini, Claudia Gemelli, Gian Antonio Danieli, Sergio Ferrari

**Affiliations:** 1 Department of Biomedical Sciences, University of Modena and Reggio Emilia, via G. Campi 287, 41100, Modena, Italy; 2 Department of Biology, University of Padova, via G. Colombo 3, 35131, Padova, Italy; 3 Department of Chemical Engineering Processes, University of Padova via F. Marzolo 9, 35131, Padova, Italy

## Abstract

**Background:**

Human myelopoiesis is an exciting biological model for cellular differentiation since it represents a plastic process where multipotent stem cells gradually limit their differentiation potential, generating different precursor cells which finally evolve into distinct terminally differentiated cells. This study aimed at investigating the genomic expression during myeloid differentiation through a computational approach that integrates gene expression profiles with functional information and genome organization.

**Results:**

Gene expression data from 24 experiments for 8 different cell types of the human myelopoietic lineage were used to generate an integrated myelopoiesis dataset of 9,425 genes, each reliably associated to a unique genomic position and chromosomal coordinate. Lists of genes constitutively expressed or silent during myelopoiesis and of genes differentially expressed in commitment phase of myelopoiesis were first identified using a classical data analysis procedure. Then, the genomic distribution of myelopoiesis genes was investigated integrating transcriptional and functional characteristics of genes. This approach allowed identifying specific chromosomal regions significantly highly or weakly expressed, and clusters of differentially expressed genes and of transcripts related to specific functional modules.

**Conclusion:**

The analysis of genomic expression during human myelopoiesis using an integrative computational approach allowed discovering important relationships between genomic position, biological function and expression patterns and highlighting chromatin domains, including genes with coordinated expression and lineage-specific functions.

## Background

In recent years, the availability of the human genome sequence disclosed novel opportunities to study biological processes from a higher level perspective. The concurrent advance in bioinformatic methods, as well as in high throughput technologies for the analysis of gene expression profiles, allowed deepening the knowledge of genome structure and function. In particular, several genomic studies suggested the existence of relationships between gene expression and genomic position. The pioneering work of Caron and co-workers [1] enlightened a higher-order organization of the genome and identified regions of increased gene expression as groups of physically contiguous highly expressed genes. The analysis of non-random genomic distribution of genes and other genomic features, such as GC rich regions [2-4], showed that tissue- or organ-specific genes are often grouped in distinct chromosomal regions [5-10]. Lee and Sonnhammer [11] identified a significant tendency to cluster of genes encoding products active in the same pathway. The correlation among co-expression and co-localization of genes was also investigated and confirmed in different organisms [12]. Different bioinformatic methods were developed to identify chromosomal regions of increased or decreased expression from transcriptional data [13-20] and to find significant local enrichment of specific features in genomes [21]. Some of these methodologies based on transcriptome mapping were also successfully used to study the correlation between expression and position of genes in tumours or in other diseases, proving to be effective both in identifying chromosomal aberrations in cancers [15-18,20] and in discovering novel genes potentially involved in tumorigenicity [22] and other disorders [23,24].

Given these experimental evidences, the integration of high-throughput transcriptional data with gene structural information and functional characteristics represents a major challenge for bioinformatics and computational biology. Indeed, an integrated approach would allow deciphering how the structural organization of genomes influences its functional utilization. For instance, the existence of tissue-specific gene clusters may be related to the efficient activation of gene expression in a particular cell lineage, by genetic and epigenetic mechanisms, or related to the repression of entire chromosomal regions containing genes expressed in a specific cell type, e.g. during the developmental switches leading to different cell lineages [25]. The analysis of transcriptome data in the perspective of the genomic organization of genes could certainly shed light not only on the aberrations leading to pathological states but also on the physiological mechanisms of all cellular processes, including cellular differentiation.

Haematopoiesis is an exciting biological model for cellular differentiation since it represents a plastic process where multipotent stem cells gradually limit their differentiation potential, generating different precursor cells which finally evolve in 8 distinct types of terminally differentiated cells [26,27]. Myelopoiesis is the part of hematopoiesis leading to differentiation of myelopoietic cell lineages (erythroid, megakaryocytic, granulocytic and mono/macrophagic). This biological process is regulated by ordered patterns of gene expression, where specific combinations of transcription factors or chromatin remodelling complexes result to be responsible for the genetic program of each hematopoietic precursor [28-30]. Many transcription factors playing a major role in the lineage choice and maturation of hematopoietic cells are known [31-33]. Several perturbation studies based on gene inactivation and ectopic expression of lineage restricted factors [31,34] highlighted the central role of gene expression regulation in governing these processes.

The present study aimed at investigating the human myeloid differentiation through the analysis of genomic data. Specifically, a bioinformatic framework was used to analyze the genomic organization and distribution of genes involved in specific differentiation lineage choice. Correlations between expression patterns of genes, their physical position and their biological roles were investigated. Moreover, this genomic approach allowed identifying, in the human genome, chromatin domains containing clusters of genes relevant for specific myeloid lineages and chromosomal regions with transcriptional activity remaining low in myelopoietic cells, which are partially overlapping with genomic clusters of genes related to non hematopoietic functions.

## Results

### The integrated myelopoiesis gene expression dataset

#### Cell types and samples

**Figure 1:**
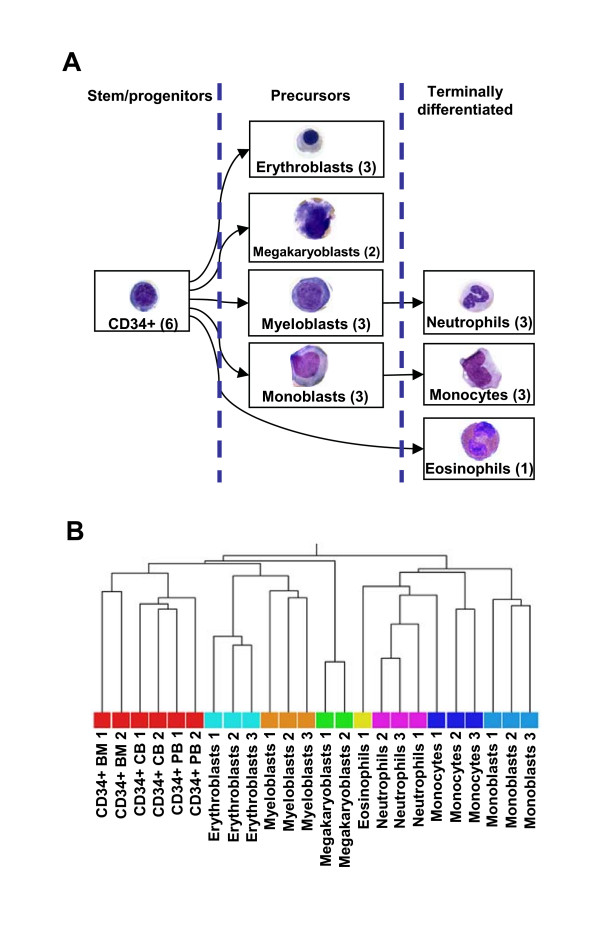
**Gene expression dataset**. A) The gene expression dataset analyzed comprises cell samples from different levels of myeloid differentiation process (stem/progenitor cells, precursors and terminally differentiated cells). The graph describes relationships between the cellular contexts analyzed within myeloid differentiation tree. For each cell type, the number of samples examined with independent microarray experiments is indicated in brackets. **B) **Dendrogram obtained by unsupervised hierarchical clustering on gene expression data matrix. Pearson correlation and average were used as similarity measure and linking method, respectively.

The original probeset-level dataset, produced using Affymetrix GeneChip HG-U133A, includes gene expression data, relative to 22,283 probesets, in 24 experiments generated from 8 different cell types of the human myelopoietic lineage: hematopoietic stem/progenitor cells (CD34+ cells), myeloid precursors (myeloblasts, monoblasts, erythroblasts and megakaryoblasts) and terminally differentiated cells (monocytes, neutrophils and eosinophils). The relationships among different cell types along the myeloid differentiation process and the number of replicates for each cell type are summarized in Figure 1A.

#### Annotation and filtering

From the original dataset, we selected only probesets reportedly associated to a unique human gene. Among them, those probeset/gene matches showing, according to the GeneAnnot database [35], either sensitivity and specificity score of 1 were chosen, thus obtaining 16,065 expression data vectors reliably associated to 11,138 different human genes. An *ad hoc *data filtering procedure was used to eliminate redundancy in expression data, i.e. multiple probesets per gene, by associating a single reliable expression vector to each EntrezGeneID. The filtered data set consisted of 10,014 gene expression vectors. Finally, when establishing the correspondence between each gene and genomic position, further 589 genes with ambiguous genomic location, were discarded. The resulting myelopoiesis expression data matrix included 9,425 genes, each reliably associated to a unique genomic position, identified as the bp interval of gene span, and to a single expression vector (Additional file 1). Although the myelopoiesis gene expression data matrix represents only a subset of all human genes, corresponding to about one half of the 18,349 known protein-coding genes reported in EntrezGene database, it's worthwhile noting that there is no significant deviation from chromosomal distribution of the 9,425 genes included in our data matrix and the chromosomal distribution of the 18,349 protein-coding human genes (p-value >> 0.05; data not shown).

### Gene expression data analysis

#### Unsupervised analysis

Unsupervised hierarchical clustering was performed to verify if the gene expression profiles of the data matrix, following the probeset annotation and filtering process described above, were able to correctly group the various phenotypes, including data from public repositories. As shown in Figure 1B, different cell types are quite well separated by unsupervised analysis performed on the whole gene expression profiles.

#### Identification of genes constitutively expressed or silent during myelopoiesis

The selection of silent and expressed genes didn't rely only on Affymetrix detection because, even if trustworthy, detection is actually only a measure of signal quality associated to each probeset, rather than an evaluation of the real transcriptional status of a gene. Therefore, in order to identify a threshold for discriminating between silent and expressed genes, we analyzed the signal values distribution of the probeset-level dataset, considering only log2 signal values corresponding to probesets with "P" detection call. The expression value of 5.44, corresponding to the 10^th ^percentile of signal values distribution, was selected as threshold. Then, this threshold was used to discriminate between genes not expressed at a detectable level, i.e. silent, and expressed genes, regardless of the detection call, either Present or Absent, associated to each gene. For each of the 8 cell types in the gene expression data matrix, we considered silent those genes showing median expression value, across replicates, lower than the threshold. Then we identified as expressed those genes with median expression value higher than the selected threshold.

Merging of the lists of genes expressed in different samples showed that a total of 5,296 genes are transcribed in every examined cell type of the myelopoietic lineage, and hence constitutively expressed during myelopoiesis (Additional file 2). Conversely, 1,418 genes resulted to be silent in all the considered myeloid cell types (Additional file 3).

**Figure 2:**
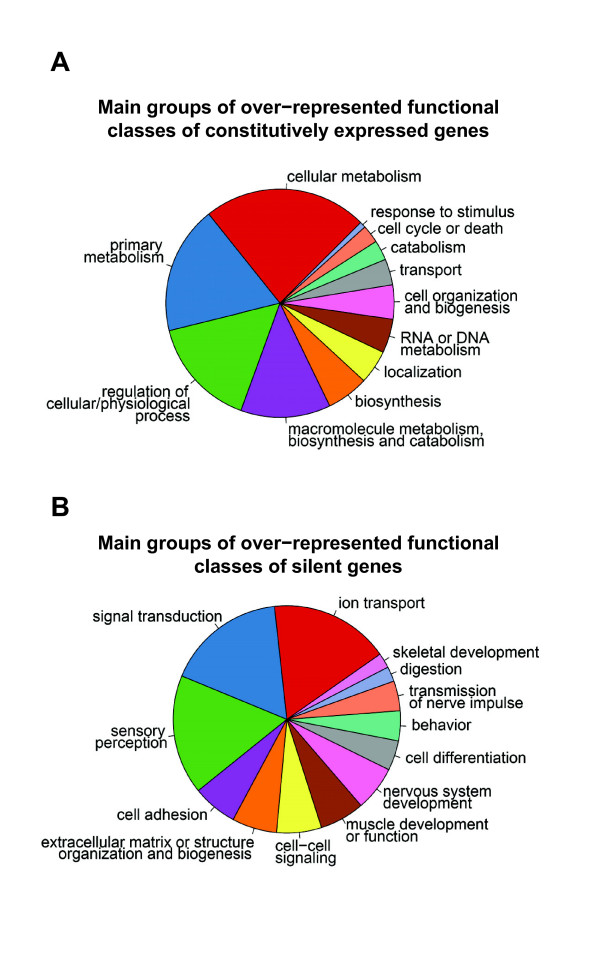
**Functional classification of constitutively expressed or silent genes during myelopoiesis**. Functional classification was performed using the functional annotation chart of DAVID 2006 and significantly over-represented Gene Ontology Biological Process categories (p-value < 0.01) were identified. Groups of functional categories were defined using a custom Gene Ontology SLIM and R script [69]. Complete lists of over-represented functional classes are reported in Additional file 2 and 3.

Functional characterization of genes constitutively expressed during myelopoiesis was attempted, by examination of biological processes involving their products, according to the functional classification of DAVID 2006 [36]. Gene Ontology terms, associated to constitutively expressed genes, and significantly more represented than expected (p-value < 0.01), refer mainly to basic cellular biological processes (energy metabolism, synthetic and catabolic metabolism of biomolecules and biopolymers); gene expression (mRNA processing, RNA splicing) and post-translational protein modification or transport; and to regulation of cell cycle or to apoptosis (Figure 2A and Additional file 2). All of these functions are necessary for basal cell activity and hence are also required by myelopoietic cells, along all of the differentiation steps, as well as by other cell types.

Significantly over-represented Gene Ontology terms, associated to silent genes during myelopoiesis, refer to biological processes related to development or function of non-hematopoietic organs or systems (Figure 2B and Additional file 3). Among these: sensory perception, transmission of nerve impulse, muscle contraction, muscle development, nervous system development, skeletal development and digestion. All of these processes are clearly not involved in myelopoiesis and it is reasonable to identify corresponding genes as "not expressed" along the entire differentiation process.

#### Identification of genes differentially expressed in commitment phase

We then focused to the commitment phase of myelopoiesis, i.e. the phase of lineage choice for the proliferating stem cells. The four different myelopoietic precursor cell types were pairwise compared with CD34+ cells, by using SAM, to identify genes differentially expressed in the commitment phase of stem cell towards each maturation process. Using fairly stringent conditions (minimum fold change = 2; number of permutations = 100 and estimated FDR < 0.01), genes significantly up-regulated and down-regulated in each lineage choice were identified (Additional file 4). The number of genes differentially expressed when comparing stem cells versus erythroblasts, myeloblasts, monoblasts and megakaryoblasts are respectively 492, 47, 403 and 350, thus showing that myeloblasts are the precursor cells most similar to CD34+ cells. It is worth note that about the two thirds of differentially expressed genes in each lineage choice are specific of a single differentiation program (e.g. 273 out of 403 genes differentially expressed in CD34+ cells versus monoblasts are specifically differentially expressed only in this comparison).

Gene Ontology terms, associated to genes significantly up-regulated in each considered commitment phase, and significantly more represented than expected (p-value < 0.01) are reported in Table 1. Enriched terms refer to peculiar biological role of each differentiation lineage. Indeed genes up-regulated in erythroblasts are mainly involved in heme biosynthesis and erythroid differentiation, or encode erythrocytes antigens (see also Table 2). Similarly, megakaryoblasts showed increased expression of genes consistent with platelet biology, being related to blood coagulation and platelet activation or to biosynthesis of steroids and particularly of thromboxanes; myeloblasts up-regulated functions related to granulocytes biology, such as immune and defense response; and monoblasts over-expressed genes involved in immune and defense response, or immune and macrophage cell activation, which are peculiar biological roles of monocytes (Table 2).

**Table 1:** Functional classification of genes differentially expressed during commitment phase

**GO biological process terms**	**p-value**	**GO biological process terms**	**p-value**
**Genes up-regulated in erythroblasts**			
heme biosynthesis	8.3593E-09	tRNA metabolism	4.7686E-04
heme metabolism	4.1178E-08	nitrogen compound metabolism	0.0011
pigment biosynthesis	7.8243E-08	cholesterol biosynthesis	0.0021
porphyrin biosynthesis	8.0158E-08	amino acid activation	0.0022
pigment metabolism	1.8805E-07	tRNA aminoacylation for protein translation	0.0022
porphyrin metabolism	2.5313E-07	tRNA aminoacylation	0.0022
carboxylic acid metabolism	3.5431E-07	electron transport	0.0023
organic acid metabolism	4.0819E-07	amino acid and derivative metabolism	0.0025
secondary metabolism	5.8601E-07	amine metabolism	0.0037
heterocycle metabolism	4.7133E-06	lipid metabolism	0.0037
cellular biosynthesis	6.9372E-06	steroid biosynthesis	0.0041
cofactor metabolism	1.0534E-05	cholesterol metabolism	0.0041
cofactor biosynthesis	1.9914E-05	iron ion homeostasis	0.0042
generation of precursor metabolites and energy	3.9831E-05	sterol biosynthesis	0.0045
cellular metabolism	5.6290E-05	mitotic cell cycle	0.0059
lipid biosynthesis	1.6546E-04	alcohol metabolism	0.0060
cellular lipid metabolism	2.3765E-04	sterol metabolism	0.0061
amino acid metabolism	4.3568E-04		

**Genes up-regulated in megakaryoblasts**			
hemostasis	0.0010	positive regulation of cell proliferation	0.0024
endocytosis	0.0011	steroid metabolism	0.0024
cellular lipid metabolism	0.0012	lipid biosynthesis	0.0026
wound healing	0.0014	regulation of receptor mediated endocytosis	0.0034
lipid metabolism	0.0020	localization of cell	0.0049
alcohol metabolism	0.0020	cell motility	0.0049
regulation of body fluids	0.0024	cytoskeleton organization and biogenesis	0.0081
transport	0.0024	cholesterol biosynthesis	0.0089

**Genes up-regulated in monoblasts**			
response to biotic stimulus	6.3071E-15	antimicrobial humoral response	3.1669E-04
immune response	2.8466E-13	detection of stimulus	6.8750E-04
defense response	7.3698E-13	protein kinase cascade	0.0010
response to pest, pathogen or parasite	1.2074E-12	wound healing	0.0014
response to other organism	2.4524E-12	intracellular signaling cascade	0.0017
response to external stimulus	1.0443E-10	positive regulation of cellular process	0.0017
response to stimulus	8.1038E-10	endocytosis	0.0019
response to wounding	1.3630E-09	I-kappaB kinase/NF-kappaB cascade	0.0025
response to stress	2.1560E-08	lymphocyte activation	0.0031
inflammatory response	3.0602E-08	blood coagulation	0.0034
cell communication	5.0323E-07	taxis	0.0035
signal transduction	7.7480E-07	chemotaxis	0.0035
organismal physiological process	8.0044E-07	innate immune response	0.0037
response to pathogen	3.1842E-06	coagulation	0.0038
humoral immune response	3.4723E-05	locomotory behavior	0.0044
detection of biotic stimulus	4.6946E-05	hemostasis	0.0045
response to bacteria	6.7200E-05	carbohydrate metabolism	0.0048
response to pathogenic bacteria	9.7266E-05	cell surface receptor linked signal transduction	0.0059
immune cell activation	1.0815E-04	positive regulation of biological process	0.0064
cell activation	1.2116E-04	macrophage activation	0.0093
humoral defense mechanism (sensu Vertebrata)	1.4837E-04	regulation of body fluids	0.0097
antimicrobial humoral response (sensu Vertebrata)	2.8361E-04		

**Genes up-regulated in myeloblasts**			
carboxylic acid metabolism	2.2728E-05	defense response	0.0018
organic acid metabolism	2.3815E-05	response to biotic stimulus	0.0025
cellular biosynthesis	7.1605E-04	immune response	0.0048
biosynthesis	0.0016	positive regulation of cell proliferation	0.0069

Gene expression data for each precursor cell type (erythroblasts, megakaryoblasts, monoblasts and myeloblasts) were compared with CD34+ cells gene expression data using SAM analysis to identify differentially expressed genes. Significantly over represented (p-value < 0.01) Gene Ontology Biological Process terms are reported for each of the examined lists of up-regulated genes.

**Table 2:** Relevant functional groups of differentially expressed genes

**Function**	**Genesymbol**	**Full name**
**Genes up-regulated in erythroblasts**		
heme biosynthesis	CPOX	coproporphyrinogen oxidase
heme biosynthesis	ALAD	aminolevulinate, delta-, dehydratase
heme biosynthesis	ALAS2	aminolevulinate, delta-, synthase 2
heme biosynthesis	FECH	ferrochelatase (protoporphyria)
heme biosynthesis	HMBS	hydroxymethylbilane synthase
heme biosynthesis	PPOX	protoporphyrinogen oxidase
heme biosynthesis	UROD	uroporphyrinogen decarboxylase
heme biosynthesis	UROS	uroporphyrinogen III synthase (congenital erythropoietic porphyria)
erythroid differentiation	GATA1	GATA binding protein 1 (globin transcription factor 1)
erythroid differentiation	TAL1/SCL	T-cell acute lymphocytic leukemia 1
erythrocytes antigens	DARC	Duffy blood group, chemokine receptor
erythrocytes antigens	CD58/LFA-3	CD58 molecule
erythrocytes antigens	EPB42	erythrocyte membrane protein band 4.2
erythrocytes antigens	ERAF	erythroid associated factor
erythrocytes antigens	CD36	CD36 molecule (thrombospondin receptor)
erythrocytes antigens	GYPE	glycophorin E
**Genes up-regulated in megakaryoblasts**		
blood coagulation and platelet activation	ADRA2A	adrenergic, alpha-2A-, receptor
blood coagulation and platelet activation	GP5	glycoprotein V (platelet)
blood coagulation and platelet activation	GP6	glycoprotein VI (platelet)
blood coagulation and platelet activation	GP9	glycoprotein IX (platelet)
blood coagulation and platelet activation	P2RY1	purinergic receptor P2Y, G-protein coupled, 1
blood coagulation and platelet activation	PROS1	protein S (alpha)
blood coagulation and platelet activation	THBS1	thrombospondin 1
blood coagulation and platelet activation	VWF	von Willebrand factor
biosynthesis of steroids	LDLRAP1	low density lipoprotein receptor adaptor protein 1
biosynthesis of steroids	TM7SF2	transmembrane 7 superfamily member 2
biosynthesis of steroids	SREBF1	sterol regulatory element binding transcription factor 1
biosynthesis of steroids	SREBF2	sterol regulatory element binding transcription factor 2
biosynthesis of steroids	CYB5R3	cytochrome b5 reductase 3
biosynthesis of steroids	VLDLR	very low density lipoprotein receptor
biosynthesis of steroids	ALOX12	arachidonate 12-lipoxygenase
biosynthesis of steroids	LTC4S	leukotriene C4 synthase
biosynthesis of steroids	PTGS1/COX1	prostaglandin-endoperoxide synthase 1 (prostaglandin G/H synthase and cyclooxygenase)
biosynthesis of steroids	TBXAS1	thromboxane A synthase 1 (platelet, cytochrome P450, family 5, subfamily A)
**Genes up-regulated in myeloblasts**		
regulation hemopoietic cells proliferation	LIF	leukemia inhibitory factor (cholinergic differentiation factor)
inflammatory response	ALOX5AP	arachidonate 5-lipoxygenase-activating protein
regulation of myeloid cells differentiation	TIMP1	TIMP metallopeptidase inhibitor 1
**Genes up-regulated in monoblasts**		
mono/macrophage activation	CD93	CD93 molecule
mono/macrophage activation	CD40	CD40 molecule, TNF receptor superfamily member 5
mono/macrophage activation	TLR1	toll-like receptor 1
mono/macrophage activation	TLR6	toll-like receptor 6
mono/macrophage activation	ICAM1	intercellular adhesion molecule 1 (CD54), human rhinovirus receptor
mono/macrophage activation	CCL2	chemokine (C-C motif) ligand 2
mono/macrophage activation	FCGR1A	Fc fragment of IgG, high affinity Ia, receptor (CD64)
mono/macrophage activation	C3AR1	complement component 3a receptor 1
mono/macrophage activation	CD163	CD163 molecule
mono/macrophage activation	IFNGR1	interferon gamma receptor 1
mono/macrophage activation	IFNGR2	interferon gamma receptor 2 (interferon gamma transducer 1)
mono/macrophage activation	CSF1R	colony stimulating factor 1 receptor
inflammatory response	IL6	interleukin 6 (interferon, beta 2)
inflammatory response	CCR1	chemokine (C-C motif) receptor 1
inflammatory response	IL1RN	interleukin 1 receptor antagonist
inflammatory response	TLR8	toll-like receptor 8
inflammatory response	PLA2G7	phospholipase A2, group VII (platelet-activating factor acetylhydrolase, plasma)
inflammatory response	SELPLG	selectin P ligand
proteins of monocytic granules	CTSB	cathepsin B
proteins of monocytic granules	CTSD	cathepsin D (lysosomal aspartyl peptidase)
proteins of monocytic granules	RNASE1	ribonuclease, RNase A family, 1 (pancreatic)
proteins of monocytic granules	RNASE6	ribonuclease, RNase A family, k6
proteins of monocytic granules	DNASE2	deoxyribonuclease II, lysosomal
fibrogenic cytokine	TGFa	transforming growth factor, alpha
dendritic cells differentiation	IL3RA	interleukin 3 receptor, alpha (low affinity)

The table reports a selected list of genes up-regulated and strictly related to peculiar biological role of each lineage. Significantly over-expressed genes were identified performing pairwise comparisons between each precursor cell type and CD34+ cells, using SAM analysis with fairly stringent conditions. This constitute a partial list of differentially expressed genes: complete lists are available in Additional file 4.

### Genomic distribution of myelopoiesis genes

#### Chromosomal distribution

**Figure 3:**
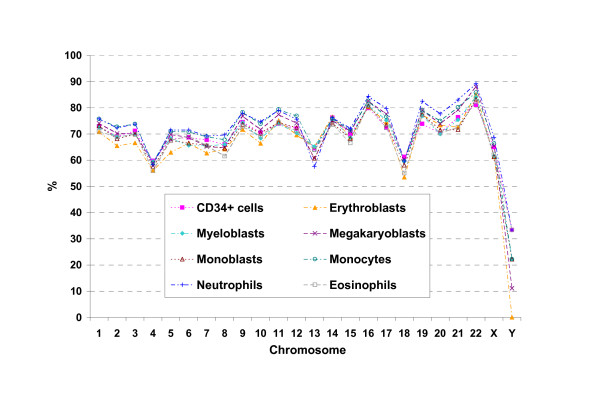
**Chromosomal expression index**. For each considered cell type the chromosomal expression index (CEI) was calculated, for each chromosome, as the percentage of genes expressed over the total number of genes considered. Across the different cell types, chromosomes 16, 19, 21 and 22 show relatively high CEI, whereas, chromosomes 4, 13 and 18 have relatively low CEI.

We first considered the distribution across chromosomes of expressed genes during myelopoiesis. For each considered cell type the chromosomal expression index was calculated as the percentage of expressed genes per chromosome (Figure 3).

Chromosomes 16, 19, 21 and 22 show high expression indexes across diverse cell types, whereas, chromosomes 4, 13 and 18 have low expression indexes.

Following these indirect indications of a possible correlation among genomic position of genes and their expression pattern during myelopoiesis, we investigated the positional clustering of genes similar for expression characteristics in the considered cell types, as well as the existence of chromosomal regions homogeneous in expression behavior.

#### Regions highly expressed identified by LAP

**Figure 4:**
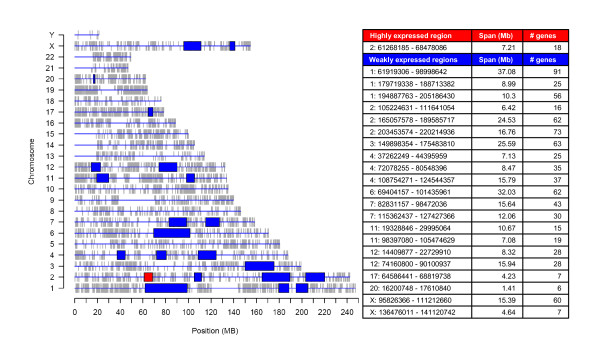
**Highly and weakly expressed regions**. The graph on the left summarizes the genomic distribution of genes included in gene expression data matrix (vertical grey lines) and points out chromosomal regions stably highly (red box) or weakly (blue box) expressed during commitment phase of myelopoiesis. In the table on the right details on the outlined regions are showed.

Using an *ad hoc *customized version of LAP (Locally Adapting Procedure[20] we searched for genomic regions significantly highly expressed. Minimum of gene expression levels across replicates was adopted as statistic for ranking probes in order of strength of expression. High peaks of the statistic, resulting significant after 100,000 permutations and with the q-value threshold set to 0.05, correspond to genomic regions with highest expression (Figure 4).

One region was found highly expressed both in CD34+ cells and in precursor cells, including 18 genes of gene expression data matrix and spanning 7.21 megabases (Mb) on chromosome 2p14-p15. The functional analysis of these 18 genes showed that most represented categories are protein transport and Golgi apparatus. The region, considering the whole set of EntrezGenes, contains 59 genes including 30 hypothetical proteins. Known genes encode proteins for basic cellular functions presumably relevant in every cell types: such as protein transport (*XPO1*, *VPS54*, *AFTPH *and *RAB1A*); amino acids glycosylation in Golgi apparatus (*B3GNT2*); Krebs cycle (*MDH1*); protein ubiquitination (*USP34*); signal transduction (*PPP3R1*); protein folding (*CCT4*) [37] and cytoskeleton structure (*ACTR2*). Finally this chromosomal area contains *MEIS1*, a regulator of proliferation and differentiation of myelopoietic precursors [38].

#### Regions weakly expressed identified by LAP

The set of genomic regions with low transcriptional activity in each considered cell type was identified by using LAP software. Maximum gene expression level across replicates was adopted as statistic and the settings of number of permutations and q-value threshold were as above. Thus, significant low peaks corresponded to genomic regions with lowest expression.

A total of 28 weakly expressed chromosomal regions, spanning 408 Mb in total, were identified in CD34+ cells. By comparing regions weakly expressed in CD34+ cells with those weakly expressed in precursor cells, we verified that 70% of these regions stably show low transcriptional activity during commitment phase of myelopoiesis: 21 regions are stably silenced, spanning 288 Mb and containing in total 788 genes (Figure 4). In addition, these regions stably silenced in commitment phase seem to be stably silenced also during terminal differentiation, because 85% of them are also weakly expressed in differentiated cells i.e. monocytes, neutrophils and eosinophils (data not shown).

The comparison of regions weakly expressed in CD34+ cells with the non redundant list of 1,055 genes differentially expressed in commitment phase, generated by merging previously mentioned lists of differentially expressed genes, showed that 89 genes differentially expressed (8.4%) fall within CD34+ weakly expressed regions. This value is significantly lower than expected by chance, as calculated using hypergeometric distribution (p-value = 0.002).

Then, the whole set of human EntrezGenes was considered, independently to the fact that they were or not represented in the myelopoiesis data matrix. Accordingly to Gene Ontology classification, human genes were associated to specific functional modules. A functional module was defined as a specific biological process or a group of functionally related biological processes: one or more Gene Ontology terms were used to filter EntrezGenes in order to define functional modules genes (see Additional file 5 for details). Genes pertaining to 8 functional modules related to development or function of non-hematopoietic organs were identified: embryonic development, epidermis development, eye development, muscle development and function, nervous system development, skeletal development, spermatogenesis, visual perception.

For each non-hematopoietic module we identified, by using REEF (REgionally Enriched Features) [21], positional enrichment of module-related genes (sliding window = 1 Mb; window shift = 200 KB; q-value cut-off = 0.05; min number of genes in cluster = 2) (Additional file 5).

A comparison of these clusters with regions stably weakly expressed during commitment phase of myelopoiesis, identified with LAP, showed overlap with clusters related to functional modules of muscle development or function, nervous system development, skeletal development and visual perception (Additional file 5).

We then focused on three peculiar genomic regions identified as stably expressed at low level by LAP (q-value < 0.01) and partially overlapping functional modules clusters identified by REEF (Additional file 5). Complete lists of human EntrezGenes included in these regions were retrieved (Additional file 6). The first region, located on chromosome 2q24-q31 (spanning 1.29 Mb), contains only 16 genes: among them 1 hypothetical protein; 1 gene of visual perception functional module (*BBS5*); *LRP2*, that could have a role in development of non hematopoietic organs [39]; sarcosin (*KBTBD10*), involved in muscle contraction [40]; *ABCB11 *and *G6PC2 *relevant in hepatocytes respectively for bile salt transport and gluconeogenesis. A second region is on chromosome 12q21 (spanning 9.36 Mb) and contains 37 genes including 9 genes for hypothetical or unknown function proteins; 2 genes of muscle development functional module as indicated by Gene Ontology (*MYF5 *and *MYF6*); *CART1*, that is involved in muscle and skeletal development [41]; and synaptic transmission genes (*NTS *[42], *SYT1 *[43] and *LIN7A*/*Veli *[44]). Finally, the third region, on chromosome 2q33-q34 (spanning 8.41 Mb), contains 58 genes including 26 genes for hypothetical or unknown function proteins: genes belonging to functional modules of nervous system development (*ADAM23 *[45]) and of visual perception (gamma-crystallins *CRYGA *and *CRYGD *that are structural constituent of crystallin) as indicated by Gene Ontology. Even if Gene Ontology classification wasn't able to highlight their functional correlation, this region contains further genes involved in non-hematopoietic functional modules, such as neurogenesis (*ERBB4 *[46], *CREB1 *[47] and *MAP2 *[48]); and other gamma-crystallins related to visual perception (*CRYGB*, *CRYGC *and pseudogenes *CRYGEP1 *and *CRYGFP1*).

#### Clusters of differentially expressed genes

**Figure 5:**
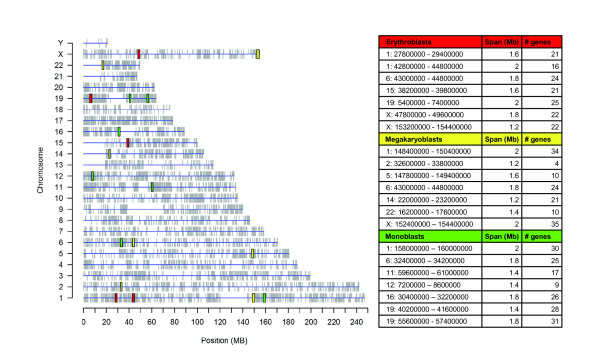
**Clusters of differentially expressed genes**. The graph on the left summarizes the genomic distribution of genes included in gene expression data matrix (vertical grey lines) and points out clusters of significantly up-regulated genes in a specific precursor cell type versus in comparison with CD34+ stem/progenitor cells: erythroblasts (red box), megakaryoblasts (yellow box) and monoblasts (green box). In the table on the right details on the outlined regions are showed.

Genomic distribution of differentially expressed genes in each specific lineage of differentiation as compared with CD34+ cells was analyzed by REEF (Additional file 7). Significant clusters were identified when analyzing most of the differentially expressed gene lists (sliding window width = 1 Mb, shift = 200 Kb, minimum selected features per clusters = 3 and q-value cut-off = 0.05)(Table 3 and Figure 5). The number of gene expression data matrix genes, included into clusters of significantly up-regulated (or down-regulated) genes and showing higher (or lower) expression level in corresponding precursor versus CD34+ cells, results to be significantly higher than expected by chance (p < = 0.01), accordingly with hypergeometric distribution (Table 3). Thus there is a common trend in gene expression within clusters identified from significantly differentially expressed genes. Furthermore, the whole set of EntrezGenes included into these regions was considered and their functional classification evaluated using DAVID 2006 [36] to identify over-represented Gene Ontology biological process terms. The most interesting functional classes are found among functional categories within genes of up-regulated clusters in monoblasts: they are clearly consistent with biological role of mono/macrophagic lineage and include classes related to immune and defense response, antigen processing and presentation, response to pathogens and cell motility (data not shown). Deeper examination of clusters of genes up-regulated in monoblasts (Figure 5) revealed some chromatin domains possibly related to monocytic function that were identified as chromosomal regions showing a good correlation among biological function, genomic position and gene expression pattern. As example we can consider the first cluster of up-regulated genes in monoblasts: this cluster, on chromosome 1q23 (spanning 2 Mb), contains 5 genes significantly up-regulated in monoblasts as compared with CD34+: among them *HSPA6 *(*HSP70B*) [49], regulating monocytes maturation towards dendritic cells; *FCER1G *[50], IgE receptor subunit expressed also in monocytes; *SLAMF8 *(*BLAME*) [51] and *SLAMF1 *[52], that are involved in lymphocytes activation. This genomic region contains a total of 73 EntrezGenes including 11 hypothetical proteins; eight IgG receptors (*FCGR2A*, *FCGR2B*, *FCGR2C*, *FCGR3A*, *FCGR3B*, *FCRLM1*, *FCRLM2*, *FCRL6*); and other antigens, belonging to SLAM family, that are involved in regulation of leucocytes activity (*CD48 *[53]; *CD84 *[54]; *CD244*, *SLAMF9*, *SLAMF6*, *SLAMF7*, *Ly9 *[55]). More genes related to mono/macrophagic functions can be found also within the other clusters of up-regulated genes, such as the genes of *MHC class II*, involved in antigen presentation.

**Table 3:** Clusters of differentially expressed genes

		**Clusters**				
**Differential expression VS CD34+ cells**	**Total number of differentially expressed genes**	**Number of clusters**	**Total number of differentially expressed genes in clusters**	**Total number of data matrix genes in clusters**	**Total number of EntrezGenes in clusters**	**p-value**

Erythroblasts up-regulated genes	269	7	26	151	360	7.94E-05
Erythroblasts down-regulated genes	223	4	14	54	127	0.0088
Megakaryoblasts up-regulated genes	214	7	25	138	379	0.0035
Megakaryoblasts down-regulated genes	136	1	3	7	18	0.0033
Monoblasts up-regulated genes	277	7	24	166	439	0.0101
Monoblasts down-regulated genes	126	0	0	0	0	nd
Myeloblasts up-regulated genes	32	0	0	0	0	nd
Myeloblasts down-regulated genes	15	0	0	0	0	nd

REEF software was used to analyse the genomic distribution of differentially expressed genes and to find significant positional enrichments (q-value < 0.05). Columns 2 and 3 indicate the total number of differentially expressed genes in the human genome and the number of clusters identified by REEF. In the following three columns, the numbers of differentially expressed genes, genes represented in the myelopoiesis data matrix and EntrezGenes falling in identified clusters are reported. The numbers of genes represented in the myelopoiesis data matrix showing up- or down-regulation, respectively for clusters of significantly up- or down-regulated genes, results to be higher than expected by chance, accordingly with hypergeometric distribution (p < = 0.01). Calculated p-values are reported in the last column. Further details concerning each cluster are reported in Additional file 7.

## Discussion

Although myelopoiesis has been extensively studied, there are still many unclear aspects, especially concerning the genetic control of this process. In the present work, normal human myelopoiesis was analyzed at the genomic level, coupling gene expression data with advanced bioinformatics tools and knowledge on sequence and annotation of the human genome. Specifically, information on gene transcription, function and genomic localization were integrated and analyzed to unravel relationships between the organization and utilization of the genome and the control of the transcriptional machinery during the differentiation process of the hematopoietic stem cell. Apart from its biological relevancy, myelopoiesis represents a perfect model system for the application of a novel computational framework able to integrate multiple types of genomic data. Indeed, hematopoietic cells have the advantage to grow in suspension as isolated, while most tissues are normally contaminated by different and non tissue-specific cell types. This growing modality allows obtaining hematopoietic samples characterized by high purity, a crucial characteristic to generate reliable gene expression data. Even though precursor cells were obtained by means of *in vitro *differentiation of hematopoietic stem cells, differentiation protocols have been deeply tested and standardized by our laboratory in order to obtain precursors with biological characteristics and differentiation potentials equivalent to *in vivo *cells: indeed they preserve the same capability to generate terminally differentiated cells [34,56-58].

A meta-analysis approach allowed integrating proprietary and public available gene expression experiments and to construct a fairly broad dataset of transcriptional data for different cell types, representing distinct lineages and distinct steps of myeloid differentiation. In particular, raw microarray data (Affymetrix .CEL files) were obtained for a total of 24 samples from 8 different cell types of all myelopoietic lineages, representing a reference dataset for a comprehensive analysis of genomic expression during cell differentiation. Since sample size is a critical issue in all microarray studies, stringent cut-offs were used in every statistical analysis that was performed, in order to strengthen the analytical results. To further enrich the number of sample replicates, public repositories of microarray data were also searched to identify high quality raw data obtained using the same Affymetrix GeneChips. Unfortunately, only few samples resulted to be biologically comparable to the proprietary data and, in particular, we could not identify additional samples concerning precursor cells. All samples were analyzed using the same generation of Affymetrix microarrays, i.e. HG-U133A, thus exploiting the high reliability and reproducibility of Affymetrix chips [59,60] and avoiding possible biases due to platform comparisons. Since the adopted approach involved mapping of transcriptional data on the genome and the analysis of connections between gene expression and gene position, the annotation of the microarray probesets has been taken into serious consideration. Although precise and regularly up-dated, Affymetrix probesets annotation has been demonstrated to present some criticalities [35,61]. Therefore, we applied an *ad-hoc *annotation procedure exploiting the information of the GeneAnnot database, in which the quality, reliability, and annotation of each probeset is quantified in terms of specificity and sensitivity scores. To strengthen our approach, we decided to select for further analysis only those probesets associated by GeneAnnot to a single gene with the maximal specificity and sensitivity, i.e. 1. Furthermore, we considered the issue of probeset redundancy, combining the expression data when multiple probesets were annotated to the same gene. Specifically, a jackknife procedure, applied to the signals of multiple probesets associated to the same gene, removed outlying probeset and generated a unique expression value only from highly correlated probesets. The pre-processing steps resulted in a gene expression matrix, composed of high-quality gene expression data for 9,425 well annotated genes. Although reduced in size as compared to the number of probes contained in the Affymetrix array, the genes represented in the myelopoiesis data matrix are a representative and comprehensive sample of all human genes.

An unsupervised hierarchical clustering of the re-annotated data matrix indicated that the integrated gene expression data contain information to separate different cell types in accordance with their morphological and functional classification, also suggesting that the inclusion of data from publicly available repository didn't cause any bias in the dataset (see Fig. 1B).

A classic supervised analysis of gene expression data was carried out to select genes constitutively expressed or silent during myelopoiesis and to identify, using SAM, genes differentially expressed during commitment phase of different lineages. The functional classification of selected gene lists confirmed the current biological knowledge and helped shedding light on myelopoiesis. Indeed, over-represented functional classes of constitutively expressed genes are mainly basic cellular functions, expected to be active in each cell type, such as metabolism or gene expression related functions. Furthermore silent genes are mainly functionally classified in development or function of non-hematopoietic organs or tissues, as nervous system, muscle or skeletal development, synaptic transmission and muscle contraction. A similar approach was also applied to the analysis of differential gene expression during commitment phase of each myelopoietic lineage. The functional classification of differentially expressed genes identified interesting peculiarities of each cell lineage. Erythroblasts showed up-regulation of heme biosynthesis genes, together with other erythrocytes antigens and genes involved in erythroid differentiation; megakaryoblasts up-regulated many genes relevant for blood coagulation and platelet function, whereas in monoblasts genes important for mono/macrophage activation and function, involved in inflammatory response and coding for monocytic granules proteins, were over-expressed. Finally, the expression profiles of myeloblasts and CD34+ resulted less divergent, consistently with previous biological evidences proving that myeloblasts are the precursor cells most similar to the hematopoietic precursor/stem cells [56]. All these results should facilitate the identification of specific genes and novel pathways relevant for lineage differentiation.

The integration of gene expression signals and structural and functional genomic information allowed the application of different bioinformatics approaches to identify and characterize higher-order organization of genomic expression during commitment phase of myelopoiesis, and to shed light on the complex network of biological mechanisms of lineage choice. We applied two procedures to upgrade the information content of transcriptional data through the analysis of chromosomal distribution of expression and functional features. In particular, a modified version of LAP approach [20] was employed to search for genomic regions characterized by consistently high or low transcriptional levels during the commitment phase, while REEF software was used to identify chromosomal regions enriched in differentially expressed genes.

LAP selected a single highly expressed and 21 weakly expressed chromosomal regions, which are stably silenced in all phases of commitment. It is noteworthy that the 70% of regions weakly expressed in CD34+ cells are stably low transcribed during commitment and include a number of differentially expressed genes statistically lower than expected (p-value = 0.002). It is further noteworthy that 85% of these regions are weakly expressed also in terminally differentiated cells (data not shown). These results support the hypothesis that there might be a positional regulation of gene expression, along myelopoiesis, accounting for gene silencing patterns that correlate with genomic position. In addition this positional regulation seems to be stable from stem cells towards more differentiated cells.

A consistent overlap was found among weakly expressed regions highlighted by LAP and clusters of genes related to functional modules silenced during myelopoiesis, identified using REEF in association with Gene Ontology annotation. Indeed, the transcriptome mapping analysis was performed on gene expression data matrix, representing a subset of genome, while clusters of functional module genes were identified among the whole set of genomic genes. Deeper analysis was performed on a restricted number of weakly expressed regions overlapping clusters of functional modules genes, selected by lowering the q-value threshold to 0.01. Within these regions we found a number of genes functionally related to non-hematopoietic organs (such as synaptic transmission, neurogenesis or visual perception genes) that could not previously be identified by classical Gene Ontology terms enrichment analysis.

The application of REEF software to the genome level analysis of the distribution of differentially expressed genes during commitment phase allowed the identification of clusters of differentially expressed genes, each involved in a specific lineage differentiation process and representing a potential biologically relevant chromatin domain. In particular, results concerning monoblasts are reported in details and are remarkably interesting, because they suggest a clear association between cluster regions of up-regulated genes and biological functions related to defense immunity, as well as overall common trend in gene expression within these regions, thus supporting the hypothesis that they could be chromatin domains relevant for monocytic function. Even if it was at least partially known that some of these genes are organized as a cluster on the genome, it is noteworthy that these regions have been identified through the computational approach used for the analysis of gene expression data. Then other genomic regions, relevant both for myelopoiesis and other biological processes, could be identified by means of the same integrated computational approach.

The identification of individual chromosomal regions stably silenced during myelopoiesis, partially overlapping with specific chromosomal areas containing genes devoted to non-myelopoietic functions, and of a number of chromosomal regions containing clusters of genes regulated in a lineage-dependent manner, seems to support the existence of relationships among structural and functional characteristics of the genome. In particular, important relationships between expression, biological function and genomic position of genes, and the presence of biologically relevant "chromatin domains" were identified in myelopoiesis, which can be considered a model system for cell differentiation. This is clearly exemplified by the fact that clusters of silent genes are mainly stable during differentiation and contain genes involved in development or function of non-hematopoietic organs, thus indicating the involvement of positional gene expression regulation in maintaining tissue specific patterns of expression. We also might suppose the involvement of epigenetic mechanisms in regulating transcription from contiguous genetic loci and that epigenetic events could account for directing CD34+ cells differentiation capabilities, with finer mechanisms involved in regulating differentially expressed genes. The data reported so far show that the analysis of gene expression profiles and functions in the context of genomic position could success in identifying and characterizing particular genomic regions presenting correlations between gene function and expression. However, further studies are required to better characterize mechanisms governing positional regulation of gene expression.

## Conclusion

This work presents a genomic approach applied to the analysis of gene expression profiles during myeloid differentiation, which substantiated the existence of relationships between genomic position, biological function and expression patterns of genes. These correlations have been demonstrated through the identification of chromatin domains including genes with coordinated expression, relevant for specific lineages function in the context of myelopoiesis.

An important result of the study was the collection of gene expression profiles describing transcriptomes of myeloid differentiating cells, providing the most comprehensive dataset covering all lineages and including both stem/progenitor, precursor and terminally differentiated cells. Gene expression data analysis provided relevant lists of genes involved in myelopoiesis, and in particular in the commitment phase of lineage choice.

## Methods

### Gene expression profiling of primary hematopoietic cells

Human CD34+ cells were purified from umbilical cord blood (CB) samples as previously described [56,62]: mononuclear cells were isolated by Ficoll-Hypaque gradient separation, washed twice with PBS, and then CD34+ cells separated using a magnetic cell sorting procedure (EasySep^® ^Human CD34 Positive Selection Kit, StemCell Technologies).

Additional CD34+ samples were obtained from bone marrow (BM), following the same protocol used for CB samples, or from peripheral blood (PB) as already described [63,64].

Briefly, PB hemopoietic stem and progenitor cells, were obtained from healthy donors who received recombinant human G-CSF (Lenograstim, Rhone-Poulenc Rorer, Milan, Italy), administered subcutaneously at 10 μg/kg per day for 5–6 days. Hematopoietic stem/progenitor CD34+ cell purification was then performed as above described.

Normal human erythroblasts were obtained from CB CD34+ hematopoietic progenitors cultured in IMDM (Euroclone) supplemented with 20% BIT (Stem Cell Technologies), 50 ng/ml SCF and 4 U/ml Erythropoietin (R&D Systems, Minneapolis, MN) for 8–10 days [58]. Differentiation of CD34+ cells was monitored daily by morphological analysis of May and Grunwald – Giemsa (MGG) stained cytospins and by flow-cytometric analysis of glycophorin A (GPA) surface antigen expression, using the phycoerythrin (PE)-conjugated mouse anti-human GPA monoclonal antibody (MoAb) (Becton Dickinson Systems, Mountain View, CA, USA).

Monoblasts (CD14+ precursors) and myeloblasts (CD14- precursors) were obtained by in vitro differentiation of CB derived CD34+ cells performed as already described [56]. Briefly, CB CD34+ cells were cultured in IMDM added with 20% FCS (Bio-Whittaker, Walkersville, MD, USA), in the presence of human hematopoietic cytokines: SCF (50 ng/ml), Flt3-ligand (Flt3-l) (50 ng/ml), IL-11 (50 ng/ml), IL-6 (10 ng/ml), IL-3 (10 ng/ml) and G-CSF (10 ng/ml) (all from R&D Systems, Minneapolis, MN, USA). After 7 days of culture, hematopoietic cells were analyzed, by flow cytometry, for CD14 antigen expression, estimated at about 25–30% of the entire cell population. Then monoblasts (CD14+) and myeloblasts (CD14-) cell fractions were obtained by immunomagnetic separation using the MACS technology (Miltenyi). Differentiation of CD34+ cells was monitored by morphological analysis of MGG-stained cytospins and by flow-cytometric analysis of CD34, CD38 and CD14 surface antigen expression.

Megakaryoblasts were also obtained by in vitro differentiation of CD34+ cells performed as already described [57]. Briefly, megakaryocytes were obtained from CD34+ cells cultivated in serum-free medium supplemented with 50 ng/mL SCF and 100 ng/mL thrombopoietin (TPO; Genzyme, Boston, MA) for 14–16 days and subsequently selected by means of a magnetic beads sorting procedure using monoclonal antibody directed against CD41a antigen (Dako, Milan, Italy).

Normal human monocytes were selected from the Ficoll separated peripheral blood (PB) mononuclear cells of adult samples by means of magnetic microbeads conjugated with mouse monoclonal (Mo) anti-human CD14 antibody (Ab) (Miltenyi, Auburn, CA) [34].

Human granulocytes were initially collected from cell pellets obtained by Ficoll separation of PB samples. Erythrocytes contained in cell pellets were removed by means of osmotic lysis. Neutrophils (CD16+ fraction) and Eosinophils (CD16- fraction) were then purified using magnetic microbeads conjugated to mouse Mo anti-human CD16 Ab (Miltenyi, Auburn, CA) [34].

The immunomagnetic systems used to collect primary cell populations always yielded a purity higher than 90%, as assessed by flow cytometry and morphological analysis (May and Grunwald – Giemsa staining)(Additional file 8). Total cellular RNA was isolated from 0.5–1 × 10^6 ^cells of each analyzed sample, by means of RNeasy Mini Kit (Qiagen, Valencia, CA) following manufacture's recommendations. Disposable RNA chips (Agilent RNA 6000 Nano LabChip kit) were used to determine the concentration and quality of RNA samples using Agilent 2100 bioanalyzer.

Gene expression profiles were obtained using Affymetrix HG-U133A GeneChip arrays which contain more than 22,000 probesets. RNA samples from cells of different donors were pooled to obtain 5 μg of total cellular RNA that were used for target synthesis according to the protocol supplied by the manufacturer (Affymetrix, Santa Clara, CA). Biotin-labeled cRNA was synthesized by means of Affymetrix One Cycle Target Labeling and Control Reagents Kit and subsequently controlled for quality and concentration using the Agilent 2100 bioanalyzer system. Labeled cRNA (15 μg) was fragmented as described in the Affymetrix GeneChip protocol. The fragmented cRNAs were then hybridized to Affymetrix HG-U133A GeneChip arrays for 16 hours. GeneChips were washed and stained by means of Affymetrix GeneChip Fluidics Station 450 using the instrument's standard Eukaryotic_GE_WS2 protocol with antibody-mediated signal amplification. Chips were scanned using the Affymetrix GeneChip Scanner 3000.

### Gene expression database annotation and construction

The proprietary gene expression collection of 20 samples, some of them previously described in [34,56,57,64], has been integrated with 4 publicly available samples from Stegmaier and co-workers [65], regarding monocytes (CL2002042640AA and CL2002042641AA) and neutrophils (CL2002042637AA and CL2002042638AA). Complete information on gene expression data are available at our web site [66]. Robust multi-array average (RMA) procedure was applied to the entire set of raw signals (i.e. .CEL files) in order to background adjust and normalize microarray intensities and to generate gene expression values. GCOS software and Affymetrix absolute analysis algorithm were used to evaluate signal specificity and reliability (Probeset Detection call: P, present; M, marginal; A, absent).

To link expression profiles to chromosomal locations Affymetrix probesets have been re-annotated using the genomic information available at the GeneAnnot database of Weizmann Institute (Release 13)[35]. GeneAnnot provides a revised and improved annotation of Affymetrix probes, whose assignment to GeneCardsIDs is ranked by sensitivity and specificity scores. In GeneAnnot, probesets have been related to GeneCards genes by direct sequence comparison of probes to GenBank, RefSeq and Ensembl mRNA sequences and sensitivity and specificity scores have been assigned to each probeset to gene match. After re-annotation, the analysis of myelopoiesis transcriptional data was limited to those probesets associated to a unique human gene and showing both sensitivity and specificity score of 1. Finally, each GeneCardsID was associated to the corresponding EntrezGeneID using GeneALaCart [67].

Since the computational tools to identify differentially expressed or enriched genomic regions require each chromosomal position being represented by a single expression signal, once re-annotated, the original database has been filtered to eliminate Affymetrix probeset redundancy. Thus, in the case of multiple probesets mapping to the same EntrezGeneID, a jackknifing procedure was applied to integrate different expression data vectors, pertaining to different probesets, in a single vector referring to a unique gene and to a single chromosomal position. Specifically, for any gene *g *represented by *N *probesets, the *N *vectors of median expression values was calculated for each block of *N-1 *probesets, obtained recursively excluding the *i-*th probeset (*i *= 1,...,*N*). Then, the Spearman correlation coefficient between the vector of median values and the expression vector of the excluded probeset has been calculated. Finally, the probesets with a Spearman correlation coefficient with the vector of median expression values lower than 0.5 were discarded. The remaining probesets were used to calculate a unique vector of median values associated to gene *g*.

The genomic position of each gene has been defined using the start and end coordinates of the corresponding KnownGene at the UCSC database. It has to be noted that the majority of EntrezGeneIDs resulted associated to more than one KnownGeneIDs (such as to different transcripts) and to different genomic positions. As such, in the case that the multiple genomic positions of a gene were in the same chromosome and overlapped or close to each other, the gene width was defined as the maximum genomic region covered by the different KnownGeneIDs elements pertaining to the given EntrezGeneID, and representing the coverage of the gene considered as a transcriptional unit.

The set of expression vectors associated each to a unique gene and to a well established genomic position constituted the myelopoiesis gene expression data matrix used for the subsequent bioinformatic analyses.

### Identification of genomic regions with homogeneous expression characteristics and enrichments

The final data matrix was analyzed using SAM [68] to identify genes whose expression is regulated during the myelopoiesis commitment phase, such as genes resulting to be differentially expressed in the different pairwise comparisons between hematopoietic stem cells (CD34+) and each of the four precursors cell types.

### LAP

Locally Adaptive Procedure (LAP) is a bioinformatic tool developed under R statistical environment for the identification of differentially expressed chromosomal regions, which accounts for variations in gene distance and density [20]. LAP procedure consists of three main steps: (1) computation of a statistic for ranking probes in order of strength of the evidence for an expression characteristic; (2) adaptive bandwidth smoothing of the statistic after sorting the statistical scores according to the chromosomal position of the corresponding genes and (3) application of a permutation test to identify differentially expressed chromosomal regions with a q-value correction for multiple tests. In this context, LAP was applied to scan the human genome and identify clusters of silent and of highly expressed genes during the various phases of myelopoiesis.

### REEF

REEF (REgionally Enriched Features) [21] is a software for identifying genomic regions enriched in specific features, such as a class or group of genes homogeneous for expression and/or functional characteristics.

The method for the calculation of local feature enrichment uses test statistic based on the hypergeometric distribution applied genome-wide by using a sliding window approach and adopting the False Discovery Rate for controlling multiplicity. In particular, REEF takes as input a list of reference features (RF; e.g. all genes contained in the myelopoiesis gene expression data matrix) mapped to a genomic DNA sequence, a list of selected features (SF) among the RF (e.g. a set genes specifically expressed in a given cell type), along with their genomic positions and the number and the length of chromosomes in the considered genome. Once selected the size of the sliding window and the shift between adjacent windows, the significance of regional enrichment in SF observed in each window, is calculated. Let *S *be the total number of *SF *over the entire genome, *R *the total number of *RF *over the entire genome, and *r *the number of *RF *in a given window (with *R *≥ *r *and *S *≥ *r*), then the probability of observing by chance at least k SF out of r RF in the window, is the pointwise significance of the observed numbers of SF in the window, as calculated by using the Hypergeometric Distribution. This probability is then used to calculate the corresponding q-value, which is in turn applied to select genome-wide significantly enriched regions.

In this context, REEF was used to identify regions enriched in genes differentially expressed in specific differentiation lineages or in genes encoding products involved in specific functional modules.

## Abbreviations

Bone marrow (BM); cord blood (CB); chromosomal expression index (CEI); megabases (Mb); peripheral blood (PB).

## Authors' contributions

FF, SBo, SBi, GAD and SF conceived the study. FF, SBo, SBi and SF wrote the paper. FF collected the datasets, carried out expression data and functional annotation analyses and participated in the biological interpretation of results. SBo and AC participated to gene expression database annotation and construction and to analyses with REEF. SBi and DB participated to data analysis and carried out the analyses with LAP. RZ and CG collected cell samples. All authors read and approved the final manuscript.

## Supplementary Material

Additional file 1 Myelopoiesis expression data matrix. Full list, annotations and data of myelopoiesis gene expression data matrix concerning 9425 genes obtained from pre-processing procedure. Click here for file

Additional file 2 Constitutively expressed genes during myelopoiesis. Complete list, annotations and functional classification results of genes constitutively expressed during myelopoiesis. Click here for file

Additional file 3 Silent genes during myelopoiesis. Complete list, annotations and functional classification results of silent genes during myelopoiesis. Click here for file

Additional file 4 Differentially expressed genes. Complete lists and annotations of differentially expressed genes obtained from comparing each precursor cell type with CD34+ stem/progenitor cells. Click here for file

Additional file 5 Clusters of functional modules genes. Complete lists of Gene Ontology terms used to define functional modules genes; clusters of functional modules genes identified using REEF and clusters overlapping weakly expressed regions. Click here for file

Additional file 6 Molecular anatomy of weakly expressed regions. Complete lists of genes of weakly expressed regions, overlapping clusters of functional modules genes related to development or function of non-hematopoietic organs. Click here for file

Additional file 7 Cluster of differentially expressed genes. Clusters of differentially expressed genes identified using REEF. Click here for file

Additional file 8 Cell samples details. Additional information on cell samples. Click here for file
