# Supplementary material for: Genomic expression during human myelopoiesis
Source: BMC Genomics. 2007 Aug 3;8:264. doi: 10.1186/1471-2164-8-264 (PMC2045681; doi:10.1186/1471-2164-8-264)
Supplement: Additional file 8 — Cell samples details. Additional information on cell samples. [file 1471-2164-8-264-S8.pdf]

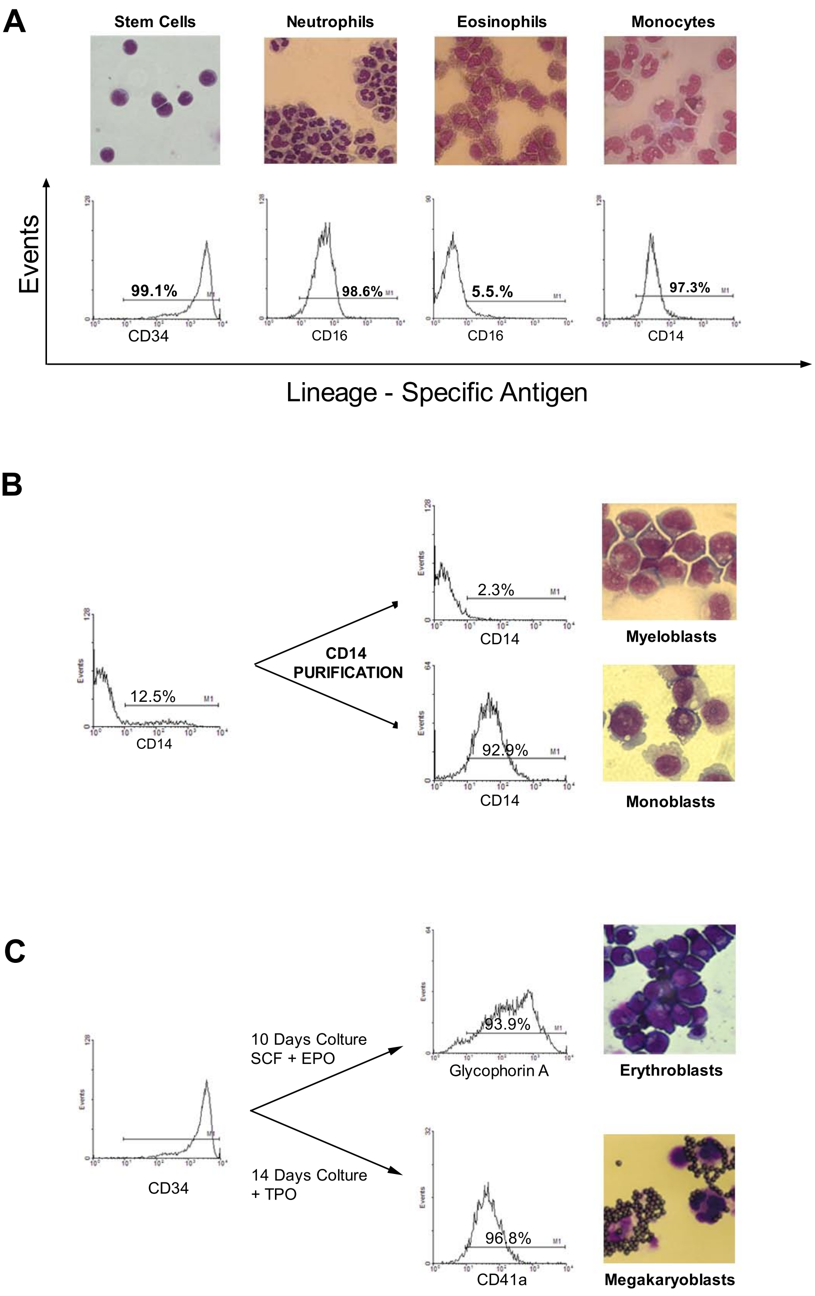

Fig. Cell samples details. Cytofluorimetric and morphological analysis of the analyzed cell populations. **A)** flow cytometry analysis of lineage specific antigens used to isolate cell populations from cord or peripheral blood. The cytofluorimetric analysis was performed after the purification procedure. In details, CD34, CD16 and CD14 antibodies have been used to positive select stem cells, neutrophils and monocytes respectively; human eosinophils were obtained as negative fraction of the CD16-based purification procedure. In the upper part of this panel a morphological analysis of each fresh population is reported. **B)** CD14+ and CD14- myeloid precursors purification and morphological analysis. In the left part of the panel, the flow cytometry analysis of CD14 expression is reported respectively in the unseparated and separated day 7 myeloid precursors. In the right side, morphological analysis of purified CD14- and CD14+ cells appears respectively as granulocyte precursors at the late myeloblast/promyelocyte of differentiation and monocyte –like precursors. **C)** flow cytometry histogram of Glycophorin A and CD41a expression which identify, respectively, erythroblasts and megakarioblasts cell populations generated in vitro from CD34+ stem /progenitors cells cultured in the presence of a proper cytokines mixture as described in the “Methods” section. Morphological analysis of cytocentrifugated preparations and May and Grunwald - Giemsa stained are reported in the right side of the panel.
